# The use of robots in nursing

**DOI:** 10.1590/1518-8345.0000.3064

**Published:** 2018-10-25

**Authors:** Maria Lúcia do Carmo Cruz Robazzi

**Affiliations:** 1 Universidade de São Paulo, Escola de Enfermagem de Ribeirão Preto, PAHO/WHO Collaborating Centre for Nursing Research Development, Ribeirão Preto, SP, Brazil.

**Figure:**
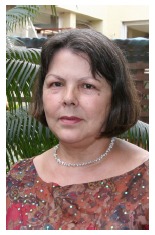

Automated devices that have feedback connections between their sensors and the environment, the robots, are an accurate and reliable technology. In the area of health, the use of robots can prevent medical errors, assist in activities such as distribution of meals and clothes in hospitals, among others.

These machines can perform procedures with high precision, reduce the duration of some techniques and facilitate the manipulation of areas of difficult access. Their use increases the possibility of curing cancer and is a well-stablished practice in developed countries. In North America, it is estimated that there are more than 3,500 robots for complex surgeries in hospital units; they adjust and compensate for trembling hands and make tiny movements with accuracy^1^.

In nursing care, the use of robots has been increasing, but modestly. Patents related to the use of robots in nursing care have been identified and their use is linked to the needs of the older adult or disabled, particularly in regions of Asia, Europe and North America^2^.

US and Egyptian nurses receive training in new technologies that use robots in surgical settings to ensure quality and safety of patient care. They participate in structured training programs that help them to gain confidence, aiming to achieve successful outcomes in the surgical procedures that involve these devices ^(^ ^3^ ^-^ ^4^. In Korea, robots were proven to be effective in nursing care services, particularly in “measuring/monitoring”. They can decrease nursing workloads, minimize care activities and are considered good co-operators in care^5^.

In Brazil, the use of robots in health has been increasing, including less invasive and painful procedures. However, their use in nursing is still restricted to training and qualification of nurses to assist in surgical procedures. Robots could be designed to perform some assistance activities, reducing work time, facilitating detailed work and assisting the professionals in providing qualified assistance.

It is believed that, soon, with careful programming, robots will be able to safely apply some dressings, accurately administer medication, perform precise changes of decubitus, assist in the transport of patients from the stretcher to the bed and from the bed to the wheelchair, and transport and administer meals and medications at previously scheduled times.

It should be emphasized that robots will never replace nursing professionals and their humanized care, but will be able to assist them in their professional practice.

The topic is innovative and thought-provoking, and progress in the scientific knowledge regarding the use of robots in professional nursing practice is still necessary. Therefore, investigations are needed for workers in this profession to use this technology appropriately and safely.
